# Alterations of synaptic plasticity in Angelman syndrome model mice are rescued by 5-HT7R stimulation

**DOI:** 10.1016/j.pneurobio.2024.102684

**Published:** 2024-10-29

**Authors:** Amelia Pizzella, Eduardo Penna, Yan Liu, Natalia Abate, Enza Lacivita, Marcello Leopoldo, Carla Perrone-Capano, Marianna Crispino, Michel Baudry, Xiaoning Bi

**Affiliations:** a Department of Biology, University of Naples Federico II, Naples, Italy; b College of Osteopathic Medicine of the Pacific, Western University of Health Sciences, Pomona, USA; c Department of Pharmacy – Drug Sciences, University of Bari Aldo Moro, Bari, Italy; d Department of Pharmacy, University of Naples Federico II, Naples, Italy; e College of Dental Medicine, Western University of Health Sciences, Pomona, USA

**Keywords:** Angelman Syndrome, Serotonin receptor 7, Synaptic protein synthesis, Dendritic spine density, LTP, Fear conditioning

## Abstract

Angelman syndrome (AS) is a severe neurodevelopmental disorder characterized by motor disfunction, seizures, intellectual disability, speech deficits, and autism-like behavior, showing high comorbidity with Autism Spectrum Disorders (ASD). It is known that stimulation of the serotonin receptor 7 (5-HT7R) can rescue some of the behavioral and neuroplasticity dysfunctions in animal models of Fragile X and Rett syndrome, two pathologies associated with ASD. In view of these observations, we hypothesised that alterations of 5-HT7R signalling might also be involved in AS. To test this hypothesis, we stimulated 5-HT7R with the selective agonist LP-211 to investigate its possible beneficial effects on synaptic dysfunctions and altered behavior in the AS mice model. In mutant mice, we observed impairment of the synaptic machinery of protein synthesis, which was reversed by 5-HT7R activation. Moreover, stimulation of 5-HT7R was able to: i) enhance dendritic spine density in hippocampal neurons, which was reduced in AS mice; ii) restore impaired long-term potentiation (LTP) in hippocampal slices of the AS mice; iii) improve cognitive performance of the mutant animals subjected to the fear conditioning paradigm. Altogether, our results, showing beneficial effects of 5-HT7R stimulation in restoring molecular and cognitive deficits associated with AS, suggest that targeting 5-HT7R could be a promising therapeutic approach for the pathology.

## 1. Introduction

The serotonin receptor type 7 (5-HT7R) is a G-protein-coupled receptor, which is the most recently discovered serotonin receptor subtype. It is broadly expressed in the central nervous system (CNS), and is involved in numerous physiological functions, including learning and memory, and synaptic plasticity (Crispino et al., 2020; Ogelman et al., 2024; Zareifopoulos and Papatheodoropoulos, 2016). 5-HT7R modulates neuronal morphology, excitability, and plasticity, contributing to shaping brain networks during development and to remodeling neuronal connectivity in adult brain (Kobe et al., 2012; Speranza et al., 2024, 2017; Volpicelli et al., 2019). Moreover, 5-HT7R’s involvement in spinogenesis and synaptogenesis, and in protein synthesis-dependent neurite and axonal elongation (Speranza et al., 2015, 2013), suggests that activation of this receptor may be linked to the axonal and synaptic machinery of protein synthesis (Crispino et al., 2014; Giuditta et al., 2008; Perrone-Capano et al., 2021).

Deregulation of 5-HT7R-mediated signalling has been implicated in numerous brain disorders (Barnes et al., 2021; Bijata et al., 2022; Fukuyama et al., 2023; Gottlieb et al., 2023; Hashemi-Firouzi et al., 2017; Quintero-Villegas and Valdés-Ferrer, 2022), including several neuropsychiatric and neurodevelopmental diseases characterized by impaired neuronal connectivity such as Autism Spectrum Disorders (ASD) (Costa et al., 2018; De Filippis et al., 2015, 2014; Khodaverdi et al., 2021). Interestingly, LP-211, a brain-permeant selective agonist of 5-HT7R (Leopoldo et al., 2011), was successfully employed to treat ASD dysfunctions in animal models of Fragile X syndrome (FXS) and Rett syndrome (RTT) (Costa et al., 2018; De Filippis et al., 2015, 2014; Napoletani et al., 2021; Valenti et al., 2017).

Angelman syndrome (AS) is a rare and incurable neurodevelopmental disorder characterized by severe intellectual disability, autism-like behavior, and seizures (Bi et al., 2016; Margolis et al., 2015; Tan and Bird, 2016). AS is due to the lack of expression in the brain of the maternally inherited *UBE3A* gene located on chromosome 15q11.2 (Tan and Bird, 2016). Neurons entirely depend on *UBE3A* expressed from the maternal allele, since the paternally inherited copy of the gene is silenced. UBE3A has been demonstrated to play a key role in neural circuit maturation and experience-dependent plasticity in the mammalian brain (Sato and Stryker, 2010; Yashiro et al., 2009). A widely used animal model of AS is the mouse strain with maternal UBE3A deficiency, which exhibits features similar to the symptoms of AS individuals such as motor dysfunction, inducible seizures, and learning and memory deficits (Jiang et al., 1998; Su et al., 2023; Sun et al., 2015b, 2015a; Van Woerden et al., 2007). AS mice also display impairment in the Long Term Potentiation (LTP) of synaptic transmission, a widely recognized cellular model of learning and memory (Baudry et al., 2012; Bi et al., 2016; Jiang et al., 1998; Kaphzan et al., 2012; Sun et al., 2018). AS has a high comorbidity with ASD, especially with regards to developmental delay and language impairment (Hogart et al., 2010; Trillingsgaard and Østergaard, 2004). Several studies have shown that UBE3A levels are crucial for determining the destiny toward AS or ASD (Furusawa et al., 2023; Khatri and Man, 2019; Lopez et al., 2019; Smith et al., 2011).

A growing body of evidence indicates impairment of the 5-HT neurotransmission system in the brain of ASD patients and in various animal models of the disease (Garbarino et al., 2019; Muller et al., 2016; Rodnyy et al., 2024; Ukkirapandian et al., 2024). However, only a few studies have directly focused on the links between 5-HT and AS. For instance, neuronal UBE3A levels may regulate 5-HT expression, resulting in altered phenotype (Farook et al., 2012). In a different study, chronic treatment of AS mice with fluoxetine, a selective serotonin reuptake inhibitor (SSRI), attenuated their molecular and behavioral deficits (Godavarthi et al., 2014). Moreover, buspirone, a partial agonist of the serotonin receptor 1A, was able to ameliorate anxiety-related behavior of AS human patients (Balaj et al., 2019). Altogether, these data suggest the involvement of the serotonergic system in the pathogenesis of AS.

In this study, we investigated how activation of 5-HT7R with its selective agonist LP-211 could affect synaptic plasticity, dendritic spine density, and learning and memory in the AS mice model. Our results demonstrated the involvement of 5-HT7R in AS pathology, which provide support for a potential pharmacological treatment of the disease using this receptor as a therapeutic target.

## 2. Materials and methods

### 2.1. Animals

Ube3A^tm1Alb/J^ mice were purchased from the Jackson Laboratory (Bar Harbor, MN). Wild-type (WT) and AS mice were obtained in-house through breeding of heterozygous females with WT males. Genotyping was carried out by polymerase chain reaction (PCR) of mouse tail DNA as previously described (Baudry et al., 2012). WT and AS pups were used for neuronal primary cultures. All animal procedures were approved by the Institutional Animal Care and Use Committee (IACUC) of Western University of Health Sciences (Pomona, California), in accordance with the National Institutes of Health guidelines for the care and use of Laboratory animals (NIH Publications No. 8023, revised 1978). Animals were kept with food and water ad libitum in a room with controlled temperature and humidity, and a 12 h light-dark regimen.

### 2.2. Hippocampal neuronal cultures

Neuronal cultures were prepared from hippocampus of WT and AS mice. Hippocampal neurons were prepared from P1-P3 mouse hippocampus as previously described (Sun et al., 2015b). Briefly, hippocampi were dissected and digested with papain (2 mg/mL, Sigma P4762) for 20 min at 37 °C. Dissociated cells were filtered with a cells strainer (Fisherbrand 22363548) and plated onto poly-D-lysine-coated coverslips in 24-well plates at a density of 10–15 ×10^3^ cells/cm^2^ in Neurobasal medium A (GIBCO 10888–022) supplemented with 5 % FBS, SM1 (StemCell Technologies), glutamine and pen/strep and kept at 37 °C under 5 % CO_2_. Whole medium was replaced with fresh culture medium (Brayn Physiology, StemCell Techonolgies 05790) supplemented with SM1 and pen/strep at DIV1 and then every 7 d. On DIV13, cells were infected with GFP lentiviral particles (SantaCruz sc-108084). On DIV18–19–20, cells were stimulated with 100 nM LP-211 (synthesized according to Leopoldo et al., 2008), 100 nM SB-269970 (Tocris 1612), or a combination of both. Cells incubated with vehicle (0.1 % DMSO) were used as control.

### 2.3. Preparation of synaptosomal fractions

Synaptosomal fractions were prepared from 3 month-old AS and WT mice, as previously described (Pizzella et al., 2023). Briefly, mice cerebral cortex (Cx) was rapidly dissected and homogenized in 9 volumes of cold isotonic medium (IM: 0.32 M sucrose, 10 mM Tris-HCl, pH 7.4), using a Dounce homogenizer. After centrifugation of the homogenate (2000 g, 1 min, 4 °C), the sediment was resuspended in the same volume of IM and centrifuged under the same conditions to yield a washed sediment containing nuclei, cell debris, and other particulates. The two supernatant fractions were combined and centrifuged at a higher speed (23,000 g, 4 min, 4 °C), to obtain a second sediment, which was resuspended in the same volume of IM and centrifuged as described above. The washed sediment, representing the synaptosomal fraction, was homogenized in IM and used for subsequent analyses.

### 2.4. SUrface and SEnsing of Translation (SUnSET)

To assess the activity of protein synthesis in AS neurons, hippocampal neurons 7th day *in vitro* (DIV7) were incubated for 30 min with 1 μM puromycin (Sigma p8833). At the end of the incubation, cells were rinsed with PBS and subsequently lysed in RIPA buffer (50 mM Tris-HCl pH 8.8, 150 mM NaCl, 0.1 % SDS, 0.5 % NP-40, 0.5 % DOC; protease and phosphatase inhibitor cocktail, Sigma-Aldrich) for western blot analysis.

Synaptosomes from cortices of 3 month-old mouse were preincubated at 37 °C for 15 min with 5 mM cycloheximide (chx, Tocris 0970) and 100 nM SB-269970, as indicated in the figures. The reaction was started by adding the synaptosomal fraction (300 μg/mL) to the incubation medium containing 20 μM puromycin, 50 mg/mL brain-derived neurotrophic factor (BDNF) (Millipore GF301), 5 mM chx, 100 nM LP-211, and 100 nM SB-269970 as indicated in the figures. After 1 h incubation at 37 °C, the reaction was stopped by cooling the samples on ice. Synaptosomes were collected by centrifugation at 23,000 g, 7 min, 4 °C. The pellet, containing synaptosomal proteins, was resuspended in RIPA Buffer, clarified by centrifugation in an Eppendorf 5415 C microcentrifuge at 14,000 rpm, 5 min, 4 °C, and stored at −80 °C for subsequent western blot analysis.

### 2.5. Gel electrophoresis and western blot analysis

Western blots were performed as previously reported (Sun et al., 2015a). Briefly, samples were lysed in RIPA buffer and the proteins concentration was determined by the BCA Protein Assay (Thermo Fisher). Proteins were denatured at 100 °C for 5 min in sample buffer (60 mM Tris-HCl pH 6.8, 10 % Glycerol, 2 % SDS, 100 mM DTT, 0.1 % bromophenol blue), resolved in 10 %-15 % SDS-PAGE and transferred to PVDF membranes (Merck-Millipore). The same amount of proteins was loaded in each lane of the gel. Membranes were blocked with 3 % BSA for 1 h, and then incubated overnight at 4 °C with antibodies against UBE3A (1:1000, BETHYL A300–351A), 5-HT7R (1:1000, Novusbio NB100–56352), GAPDH (1:1000, Millipore MAB374), and Puromycin (1:1000, Millipore 12D10), followed by incubation with secondary antibodies (IRDye secondary antibodies) for 2 h at room temperature. Antibody binding was detected using the Odyssey imaging system.

### 2.6. Immunofluorescence and morphological analysis

GFP-infected hippocampal neurons from WT and AS mice were fixed at DIV21 in 2 % paraformaldehyde (PFA)/10 % sucrose for 15 min at 37 °C. After permeabilization with 0.05 % Triton X-100/PBS for 5 min at 4 °C, cells were treated with 0.02 % Tween-20/PBS for 2 min at 4 °C. After two washes with ice cold PBS, cells were incubated 1 h at room temperature with 3 % BSA/PBS as blocking solution. Cells were then incubated with Rhodamine-Phalloidin (1:1000, Invitrogen R415) for F-actin staining, and with anti GFP antibodies (1:1000, Abcam AB290–1001) in 1 % BSA/PBS overnight at 4 °C. Coverslips were then washed twice with ice cold PBS for 10 min each and then incubated with secondary antibodies (Alexa Fluor-488 anti-rabbit, 1:200, Invitrogen A32731) in 1 % BSA/PBS for 2 h at room temperature. Coverslips were then washed four times with ice cold PBS for 10 min each and mounted on glass slides using VECTASHIELD mounting medium containing DAPI (Vector Laboratories). The staining was visualized in GFP-expressed neurons. Mean fluorescence intensity (MFI) was calculated over a specific region of interest, and background staining of the sections was measured and subtracted from the total signal to obtain the specific signal.

Images were acquired using a Zeiss LSM 880 with Airyscan confocal laser-scanning microscope with a 60 X objective. Images for all groups in each experiment were obtained using the same acquisition parameters and analyzed using ImageJ software (NIH). All immunostaining studies were performed in three independent experiments.

### 2.7. Dendritic spine analysis

The number of spines located on randomly selected dendritic branches was counted manually. Spine density was calculated by dividing the number of spines on a segment by the length of the segment and was expressed as the number of spines per μm of dendrite. About 15 different dendritic branches of 20 μm in length, derived from at least 3 different neurons, were analyzed and the results were averaged for each experiment. The analysis was performed on 3 litters for each experimental condition.

### 2.8. Electrophysiology of acute hippocampal slices

Acute hippocampal transversal slices (350 μm-thick) were prepared from 3 month-old mice according to standard methods (Kramár et al., 2006). Slices were maintained in an interface recording chamber under continuous perfusion with oxygenated (95 % O_2_/5 % CO_2_) and pre-heated (33 ± 0.5 °C) artificial cerebrospinal fluid (aCSF; 110 mM NaCl, 5 mM KCl, 2.5 mM CaCl_2_, 1.5 mM MgSO_4_, 1.24 mM KH_2_PO_4_, 10 mM D-glucose, 27.4 mM NaHCO_3_). Field excitatory postsynaptic potentials (fEPSPs) were recorded in CA1 stratum radiatum following stimulation of the Schaffer collateral pathway as previously described (Baudry et al., 2012). LTP was induced by theta burst stimulation (TBS; 10 bursts at 5 Hz, with each burst consisting of 4 pulses delivered at 100 Hz, with an interburst interval of 200 ms). Amplitudes and slopes of fEPSPs were measured and normalized to the average of the values recorded during the 10-min baseline before TBS. For each mouse brain, one or two slices were analyzed. Data were collected and digitized by Clampex.

### 2.9. Fear conditioning test

AS and WT mice were randomly assigned to either LP-211 or vehicle group and blinded to the examiner. LP-211 was dissolved in a vehicle solution (0.5 % DMSO in 0.9 % NaCl). AS and WT mice assigned to drug or vehicle group received intraperitoneal (ip) injections of LP-211 (3 mg/kg) or vehicle solution respectively, 30 min before the training session, as previously reported (Costa et al., 2018).

Mice were handled daily for 5 days before training. The fear-conditioning test was performed during the last 6 h of the light cycle. The conditioning chamber, a Plexiglas cage (29 cm × 29 cm × 29 cm) with a grid floor made by stainless steel rods (Coulbourn Instruments, Allentown, PA), was placed in a sound-attenuating box. The conditioning chamber was cleaned with 10 % ethanol to provide a background odour. A ventilation fan provided a background noise at about 55 dB. The animal behavior was recorded by a video camera system. On the first day of training, mice were placed in the chamber and, after a 2 min exploration period, three tone-foot-shock pairings separated by 1 min intervals were delivered. The tone (85 dB, 2 kHz) lasted for 30 s, and, in the last 2 s, the foot-shock (0.75 mA) was administered. The inter-trial intervals lasted 1 min. Mice remained in the training chamber for an additional 30 s after the last foot shock and then they were returned to their home cages. Twenty-four hours after training, the context test was performed. Mice were placed back into the conditioning chamber, without foot shocks or tones, and their behaviors were recorded for 5 min. Forty-eight hours after training, animals were subjected to the tone test. To perform this test, the conditioning chamber was modified by replacing the metal grid floor with a plastic sheet, white metal walls with plastic walls gridded with red tapes, and ethanol odour with acetic acid. The ventilation fan was turned off to reduce the background noise and the ceiling light was changed from yellow to white. Mice were placed in the altered chamber for an acclimatation period of 5 min during which their freezing level in the altered context was measured. After this period, a tone (85 dB, 2 kHz) was delivered for 1 min to measure their freezing level in response to tone. Mice behavior was recorded with the Freezeframe software and analysed with the Freezeview software (Coulbourn Instruments). Freezing was defined as the absence of visible non-respiratory movements of the body and vibrissae for more than 1 s. The percent of time animal froze was calculated and the group means with SEM and cumulative distribution of % freezing was analysed (Sun et al., 2015a).

### 2.10. Statistical analyses

Results are presented as means ± SEM. Data are compared by paired or unpaired Student’s t-test, RM one-way ANOVA followed by Dunnet’s post-hoc test, one-way ANOVA followed by Tukey’s post-test, or twoway ANOVA followed by Tukey’s or Bonferroni’s post-test, as indicated in figure legends. Post hoc tests were run when F was significant. Data are analyzed using GraphPad Prism 8, and results with P <0.05 are considered significantly different.

## 3. Results

### 3.1. An active system of protein synthesis is present in synaptosomes from WT and AS mice

**Fig. 1.**
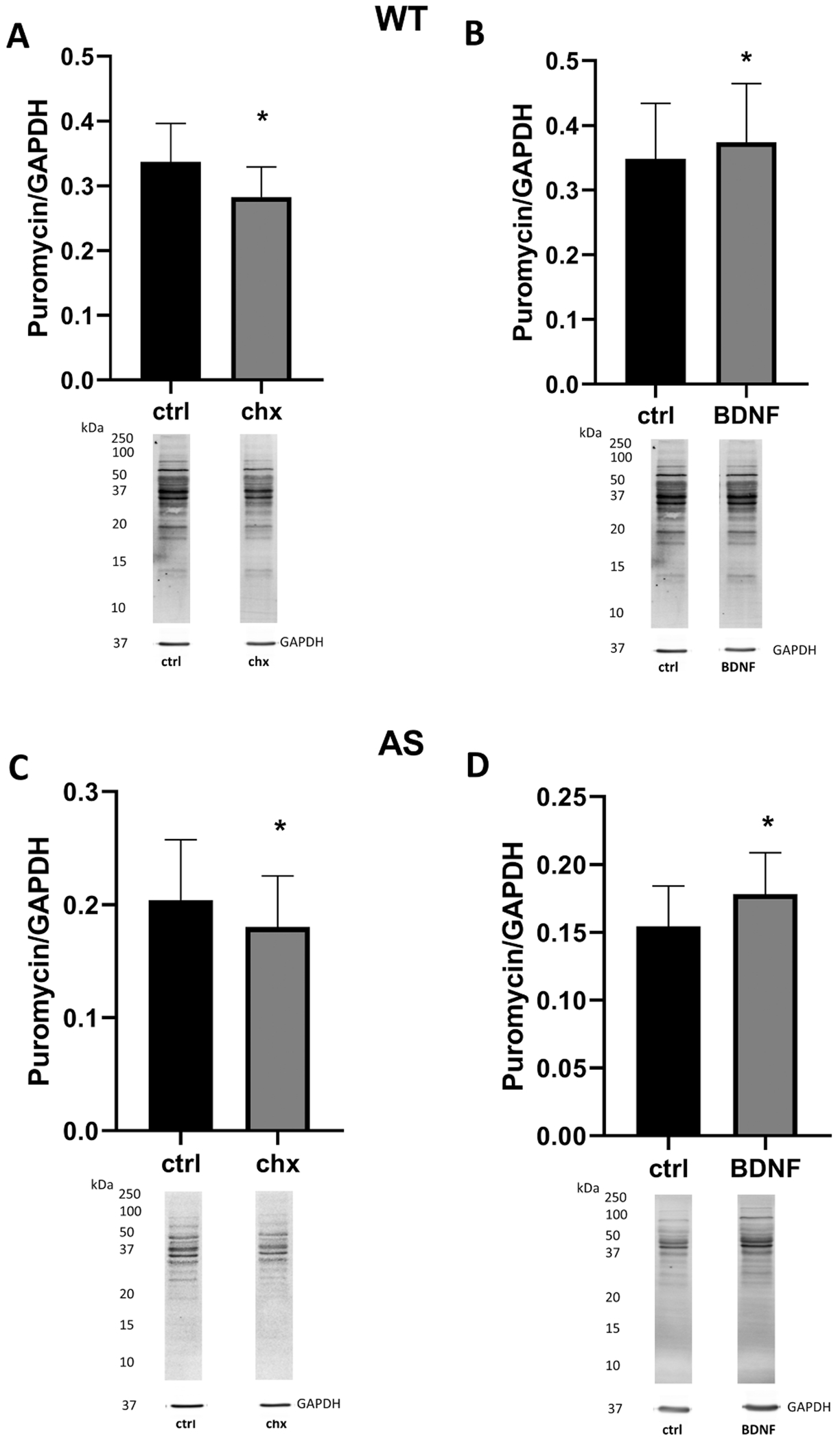
Protein synthesis in synaptosomes from cerebral cortex of WT and AS mice. Levels of puromycinilated proteins normalized to GAPDH in synaptosomes prepared from cortex of WT and AS mice, incubated respectively with **(A, C)** cycloheximide (chx), or **(B,D)** BDNF. Representative western blot images are shown below the graphs. Protein molecular weight in kDa is indicated on the left of the images. Data are presented as means ± SEM of n=5–7 mice. Statistically significant differences evaluated by t-test for paired data, * p<0.05.

Synaptic protein synthesis is a key mechanism involved in synaptic plasticity, and is impaired in numerous neuropathologies (Perrone-Capano et al., 2021), although there is no direct evidence regarding its involvement in AS. We compared the level of protein synthesis in synaptosomal fractions prepared from cortex of 3 month-old AS and WT mice. When synaptosomes were preincubated with cycloheximide, an inhibitor of eukaryotic translational elongation, a significant reduction of newly synthesized puromycin-labeled proteins was observed in both WT and AS mice (Fig. 1A,C). On the other hand, treatment of synaptosomes with BDNF, a well-known activator of protein synthesis, increased newly synthesized puromycin-labeled proteins in both WT and AS mice (Fig. 1B,D). The cycloheximide-dependent decrement, and the BDNF-dependent increment, confirmed that an active system of protein synthesis is present in both WT and AS synaptosomes.

### 3.2. Protein synthesis is selectively impaired in AS synaptosomes

**Fig. 2.**
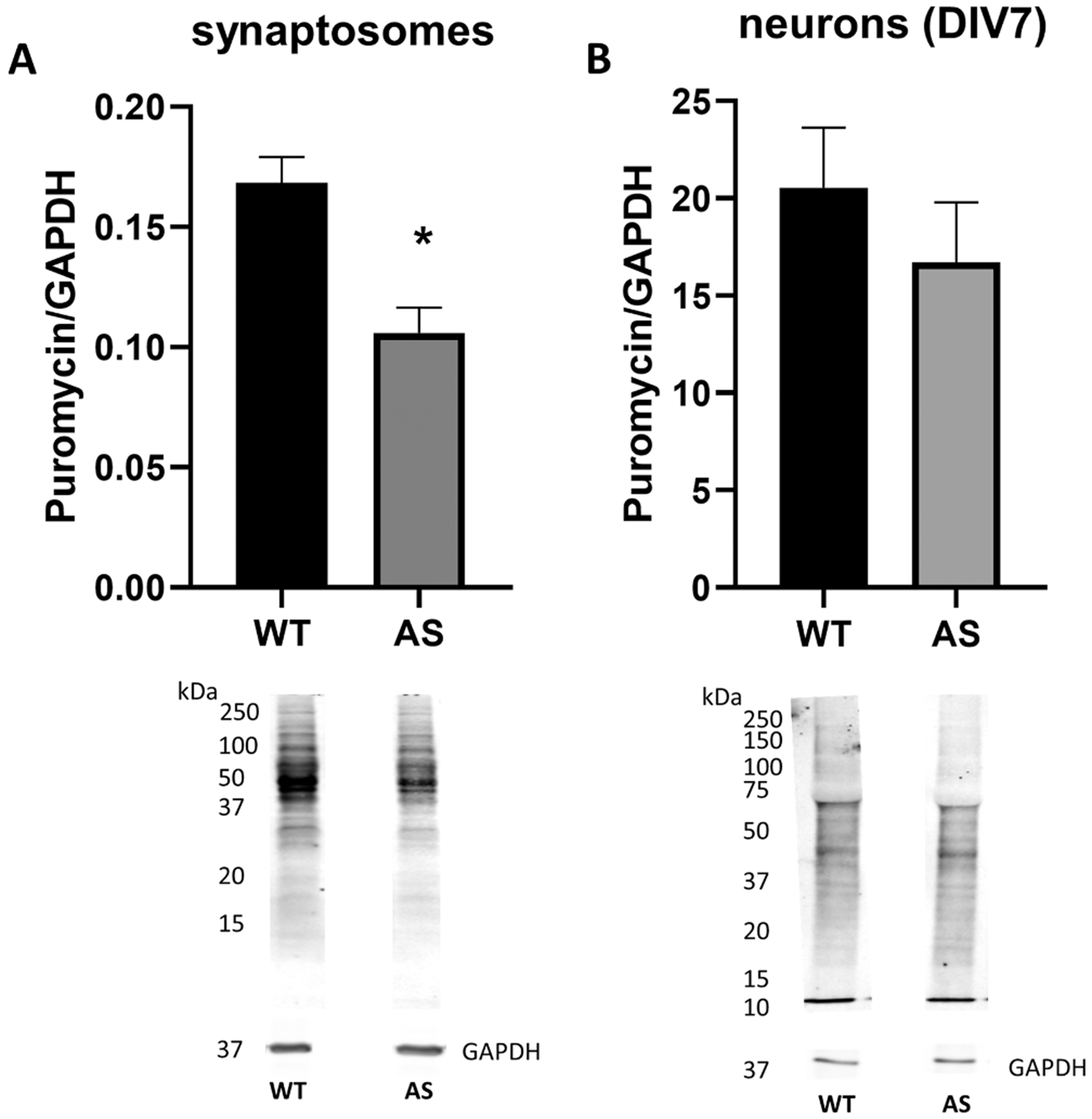
Comparison of protein synthesis in cerebral cortex synaptosomes, and in hippocampal neurons from WT and AS mice. Levels of puromycinilated proteins normalized to GAPDH in **(A)** synaptosomes prepared from cerebral cortex of WT and AS mice, and **(B)** DIV7 hippocampal primary neurons isolated from WT and AS mice. Representative western blot images are shown in the panel below the graphs. Protein molecular weight in kDa is indicated on the left. Data are presented as means ± SEM of n=3 mice. Statistically significant differences evaluated by t-test for unpaired data, * p<0.05.

Using the SUnSET labeling method we directly compared local protein synthesis in synaptosomes from AS and WT mice. Interestingly, the results showed a significant reduction of protein synthesis in synaptosomes from cortex of AS mice as compared to WT (Fig. 2A), indicating that the system of protein synthesis, operating in synaptic areas of AS mice brain, is significantly compromised.

We also evaluated the levels of neuronal protein synthesis in AS hippocampal neurons at DIV7 as compared to WT (Fig. 2B). We observed a decrease of protein synthesis in AS neurons as compared to WT, but the difference was not statistically significant. Thus, we conclude that the protein synthesis impairment in AS is likely related to the synaptic translation machinery and not to overall neuronal protein synthesis.

### 3.3. 5-HT7R stimulation differentially modulates local protein synthesis in synaptosomes from WT and AS mice

**Fig. 3.**
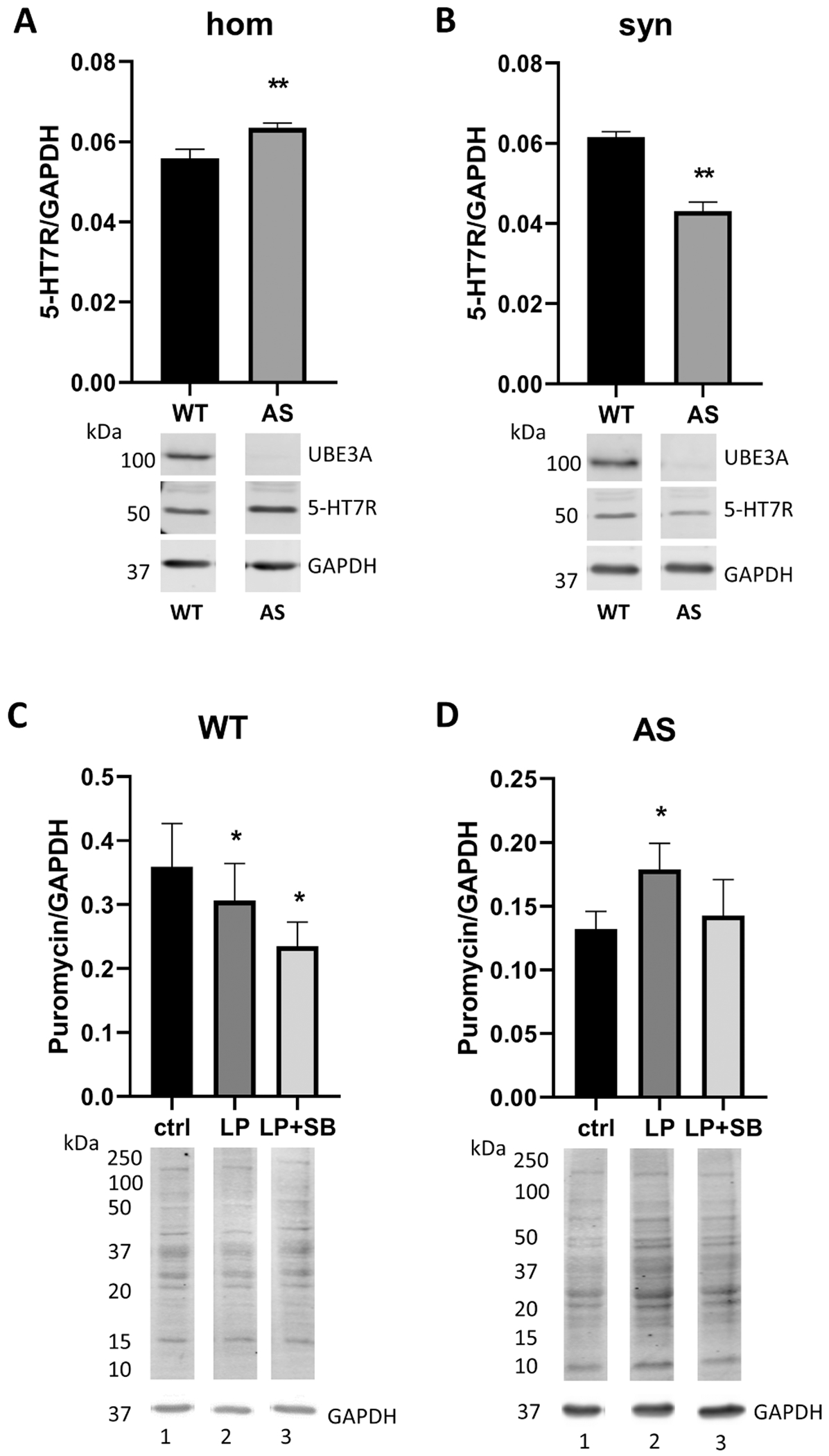
5-HT7R levels and effects of 5-HT7R stimulation on protein synthesis in synaptosomes from cerebral cortex of WT and AS mice. 5-HT7R and UBE3A levels normalized to GAPDH in **(A)** the homogenates (hom) and **(B)** synaptosomes (syn) from cerebral cortex of WT and AS mice. Levels of puromycinilated proteins normalized to GAPDH in synaptosomes from cerebral cortex of **(C)** WT and **(D)** AS mice incubated with LP-211 (LP, 100 nM) alone, or in the presence of SB-269970 (SB, 100 nM). Representative western blot images are shown below the graphs; protein molecular weight in kDa is indicated on the left. Data are presented as means ± SEM of n=5–7 mice. Statistically significant differences evaluated by **(A,B)** t-test for unpaired data, or **(C,D)** RM one-way ANOVA followed by Dunnet’s post-hoc test, comparing LP values with the corresponding control values, and LP+SB values with the corresponding control values, **(C)** F (1.148, 4.594) = 10.30, P=0.0253, **(D)** F (1.412, 8.474) = 5.283, P=0.0404. * p<0.05, ** p<0.01.

To evaluate 5-HT7R expression in synaptic terminals, we analyzed synaptosomes as well as brain cortex homogenates from which synaptosomes were isolated. 5-HT7R expression was higher in homogenates of AS mice as compared to WT (Fig. 3A), while it was reduced in synaptosomal fractions (Fig. 3B). These results suggest a shift in the distribution of 5-HT7R between different subcellular compartments in AS mice. As expected, UBE3A was mostly absent in AS homogenates and synaptosomes (Fig. 3A,B).

Stimulation of synaptosomes from WT mice with the 5-HT7R agonist, LP-211 (100 nM, 1 h), produced a decrease in levels of newly synthetized proteins (Fig. 3C). Co-incubation with SB-269970 did not prevent this decrease (Fig. 3C), suggesting that the effect of LP-211, in WT synaptosomes, was not entirely dependent on 5-HT7R. On the other hand, LP-211 treatment in synaptosomes from AS mice elicited a significant increase of protein synthesis, which was prevented by co-incubation of the agonist with the antagonist (Fig. 3D).

Therefore, it is possible to conclude that the decrease in synaptic protein synthesis in AS (Fig. 2A) is, at least partially, reversed by activation of 5-HT7R (Fig. 3D).

### 3.4. Decreased dendritic spine density in AS mice is reversed by 5-HT7R stimulation

**Fig. 4.**
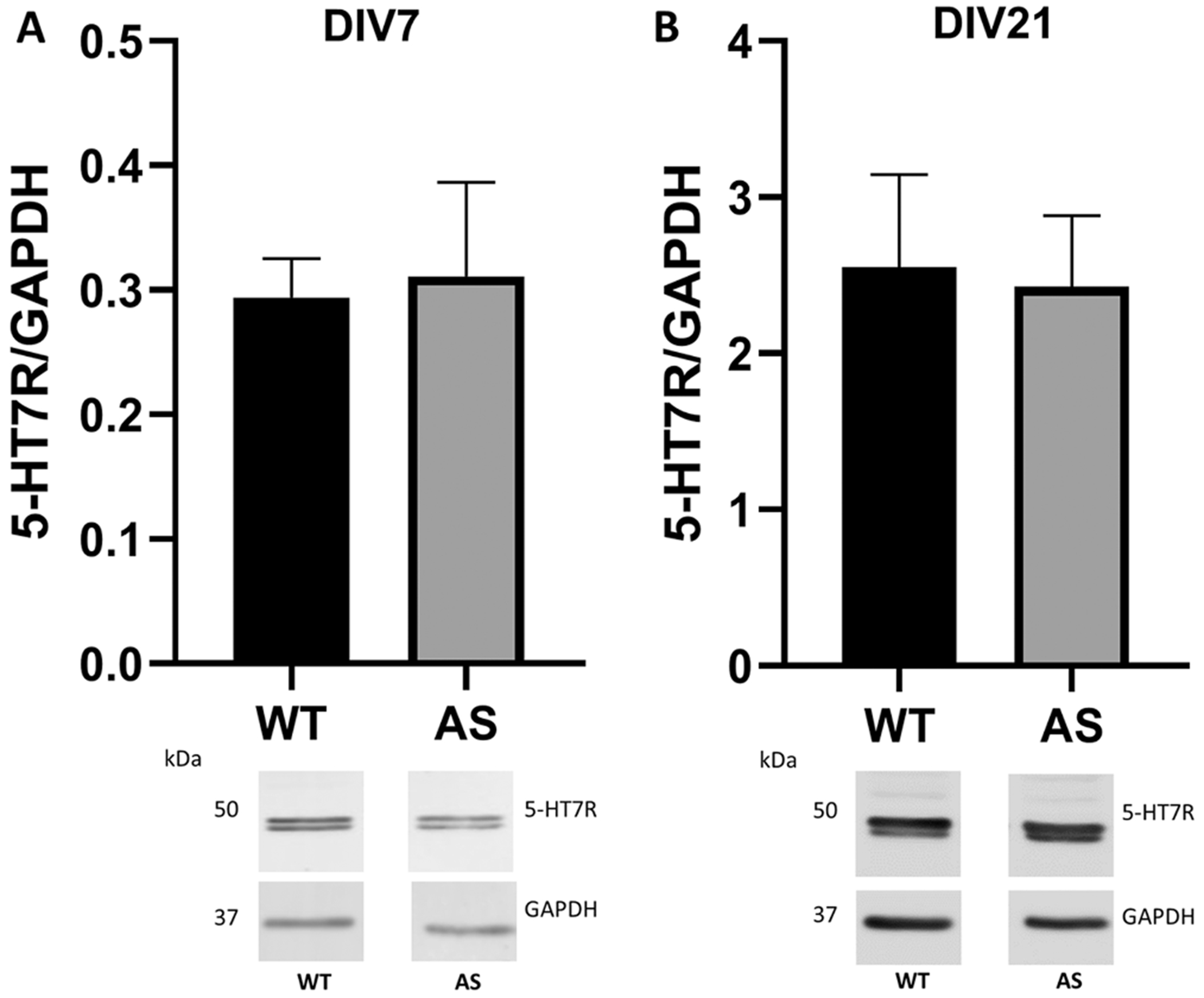
5-HT7R protein levels in hippocampal neurons from WT and AS mice. 5-HT7R levels normalized to GAPDH in the lysate from **(A)** DIV7 and **(B)** DIV21 hippocampal primary neurons from WT and AS mice. Representative western blot images are shown in the panel below the graphs; protein molecular weight in kDa is indicated on the left. Data are presented as means ± SEM of n=3 mice. Statistically significant differences evaluated by t-test for unpaired data.

We previously reported that AS hippocampal neurons exhibit a decrease in dendritic spine density as compared to WT neurons (Baudry et al., 2012). Since stimulation of 5-HT7R with LP-211 has been shown to enhance spinogenesis (Speranza et al., 2017), we tested whether LP-211 treatment could rescue the decrease in spine density in AS neurons. As a first step, we investigated, by western blot analysis, 5-HT7R expression levels in DIV7 and DIV21 hippocampal neurons prepared from P1-P3 AS and WT mice. No difference was observed between AS and WT animals, indicating that 5-HT7R expression in AS hippocampal neurons is not altered during neuronal development (Fig. 4).

**Fig. 5.**
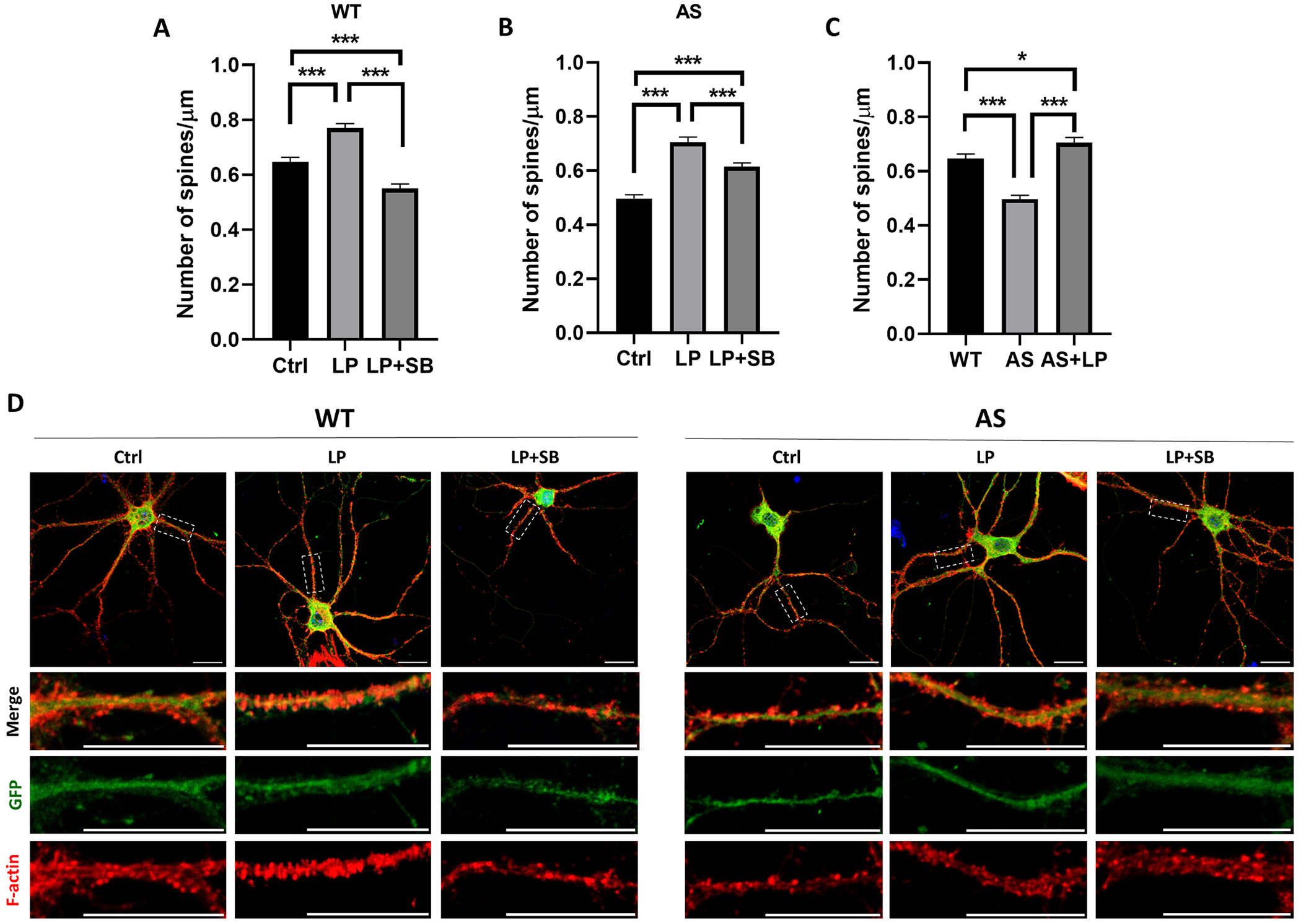
Rescuing effect of 5-HT7R stimulation on changes of dendritic spine density in AS hippocampal neurons. Dendritic spine density of GFP-infected hippocampal primary neurons at DIV21 isolated from **(A)** WT and **(B)** AS mice incubated with LP-211 (LP, 100 nM) alone, or in the presence of SB-269970 (SB, 100 nM) for 72 h. **(C)** comparison of dendritic spine density in WT and AS neurons incubated with or without LP**.** Neurons were immunostained with F-actin antibodies. n=38–50 neurons from 3 independent experiments were analysed for each group. **(D)** Representative images of data showed in **A-C**. Statistically significant differences evaluated by one-way ANOVA followed by Tukey’s post-hoc test, **(A)** F (2, 151) = 46.30, P<0.0001, **(B)** F (2, 129) = 45.86, P<0.0001, **(C)** F (2, 144) = 41.73, P<0.0001. * p<0.05, *** p<0.001. Ctrl: control. Scale bar: 20 μm.

At DIV21, hippocampal neurons from WT and AS mice were chronically stimulated (72 h) with LP-211 (100 nM), SB-269970 (100 nM) or a combination of both. Activation of 5-HT7R increased dendritic spine density in neurons from both WT and AS mice (Fig. 5A,B, D). Co-incubation of neurons with LP-211 and SB-269970 completely abolished the effect of 5-HT7R activation in neurons from WT mice, reducing the density of dendritic spines to a value even lower than in control (Fig. 5A,D). In AS mice, co-incubation of neurons with 5-HT7R agonist and antagonist only partially reduced dendritic spine density as compared to LP-211 alone, as the value remained significantly higher than in control (Fig. 5B,D). Altogether these data indicate that activation of 5-HT7R can rescue some of the neuronal morphological changes related to AS pathology (Fig. 5C,D).

### 3.5. Stimulation of 5-HT7R rescues LTP alteration in CA1 field of hippocampal slices from AS mice

We and others previously reported that TBS-induced LTP was impaired in field CA1 of hippocampal slices from AS mice (Baudry et al., 2012; Jiang et al., 1998; Liu et al., 2019; Van Woerden et al., 2007).

**Fig. 6.**
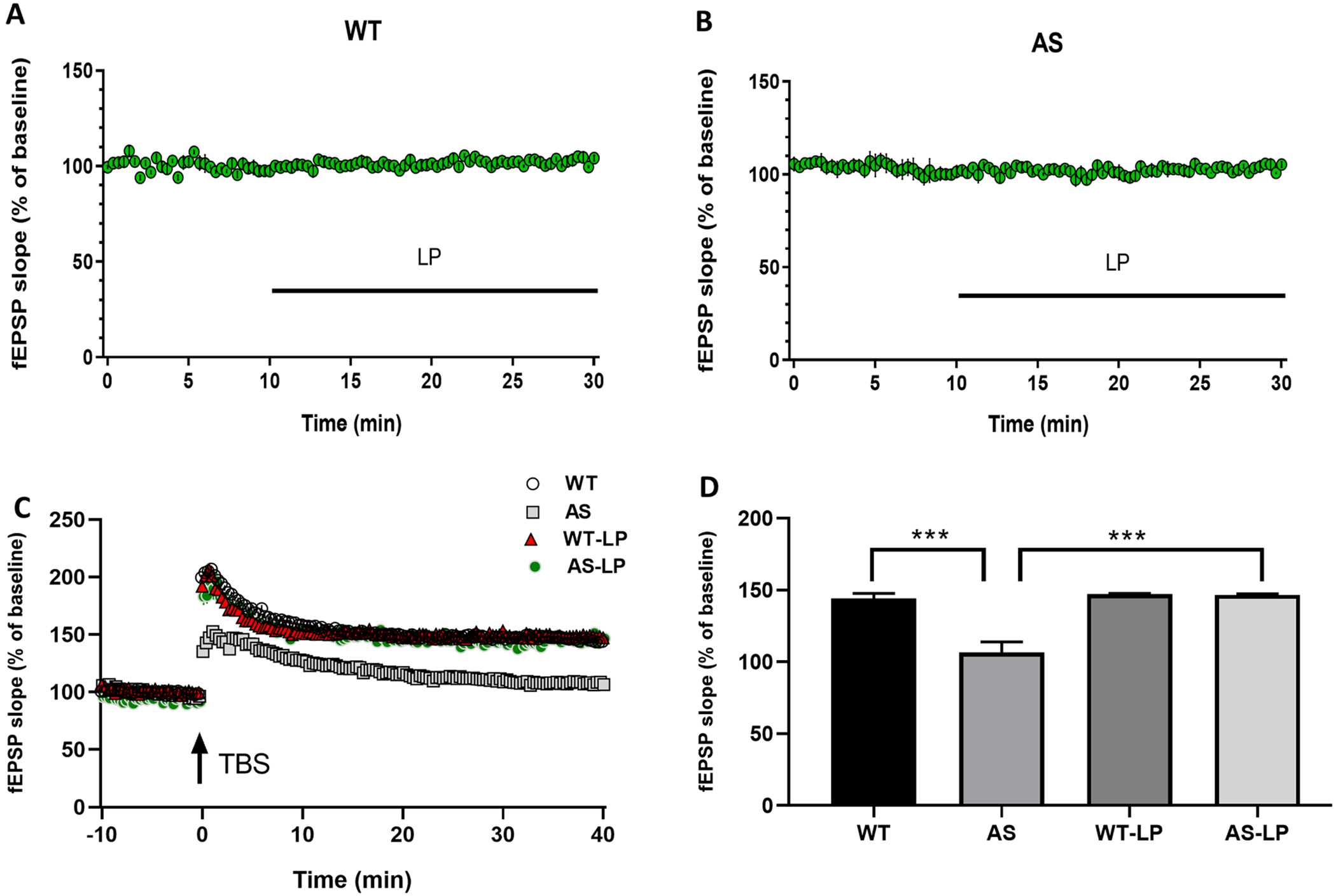
5-HT7R stimulation reverses impairment of TBS-induced LTP in field CA1 of AS hippocampal slices. Field excitatory post-synaptic potentials (fEPSP) slopes were calculated as percent of fEPSP slopes averaged over the 10 min baseline period before adding LP-211 (LP, 1 μM). **(A)** Baseline recording in CA1 field of hippocampal slices from WT mice. **(B)** Baseline recording in CA1 field of hippocampal slices from AS mice. **(C)** Acute slices from the hippocampus of WT and AS mice were treated with vehicle or LP 30 min before theta burst stimulation (TBS). fEPSP slopes were calculated as percent of fEPSP slopes averaged over the 10 min baseline period before TBS. **(D)** Long term potentiation (LTP) amplitude was measured 40 min after TBS as the percent of baseline fEPSP slopes. Data are presented as means ± SEM of values obtained in 6 slices from 3 to 5 mice. Statistically significant differences evaluated by two-way ANOVA followed by Tukey’s post-hoc test, **(D)** F (3, 26) = 18.39, P<0.0001. *** p<0.001.

In order to analyze a possible rescuing effect of LP-211 on impaired LTP in AS, we first verified that treatment of hippocampal slices with LP-211 did not affect baseline recording in WT as well as in AS mice (Fig. 6A,B). Also, we confirmed that TBS produced only short-term potentiation in hippocampal slices from AS mice, with fEPSP slope returning to baseline levels in about 30 min (Fig. 6C). Incubation of slices with the selective 5-HT7R agonist LP-211 for 30 min before TBS, resulted in recovery of TBS-induced LTP in hippocampal slices from AS mice (Fig. 6C,D). LP-211 treatment of hippocampal slices from WT mice did not modify TBS-induced LTP (Fig. 6C,D).

### 3.6. Stimulation of 5-HT7R reverses cognitive impairment in AS mice

**Fig. 7.**
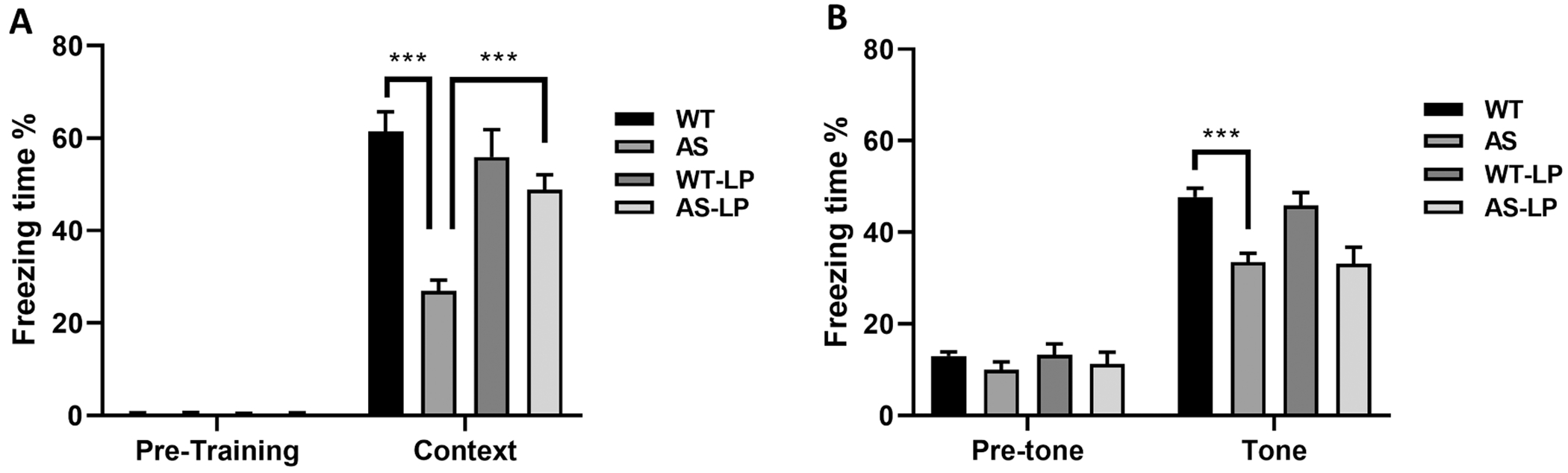
5-HT7R stimulation reverses impairment in contextual fear conditioning of AS mice. WT and AS mice were treated with vehicle or LP-211 (LP, 3 mg/kg, ip) 30 min before fear conditioning training. **(A)** % freezing time for different experimental groups in context memory. **(B)** % freezing time for different experimental groups in tone memory. Data are presented as means ± SEM of n=4 mice. Statistically significant differences evaluated by two-way ANOVA followed by Bonferroni post-hoc test, **(A)** F (3, 54) = 13.28, P<0.0001, **(B)** F (3, 54) = 7.250, P=0.0004. *** p<0.001.

We also previously reported that AS mice exhibited learning and memory impairment in both the context and tone paradigm of fear conditioning (Baudry et al., 2012; Liu et al., 2019; Sun et al., 2016). To determine whether 5-HT7R activation could rescue these deficits, WT and AS mice were injected with vehicle or LP-211 (3 mg/kg) 30 min before fear-conditioning training. In the context version of the test, AS mice treated with LP-211 exhibited a significant increase in freezing time, while LP-211 treatment did not affect freezing responses of WT mice (Fig. 7A). On the other hand, when animals were tested in the tone version of the test, the impaired performance of AS mice was not reversed by LP-211 treatment (Fig. 7B). No difference in freezing time was detected in the pre-conditioning period, or before the tone application in all experimental groups.

## 4. Discussion

Numerous lines of evidence support the involvement of 5-HT7Rs in synaptic plasticity and their participation in ASD pathology. Although AS displays several comorbidities with ASD, there are currently no reports indicating a direct link between 5-HT7Rs and AS. The goal of the present study was to investigate the potential contributions of 5-HT7Rs in AS pathology by examining the effects of 5-HT7R stimulation in the AS mouse model.

It was recently demonstrated that UBE3A activity in presynaptic terminals is required for physiological synapse elimination during postnatal development. Thus, the lack of UBE3A in AS synapses may be responsible for the prevention of synapse maturation, leading to impaired synaptic plasticity, which characterizes the disease (Furusawa et al., 2023). Considering the role of 5-HT in synapse formation and regulation, we compared the effects of 5-HT7R stimulation on a number of parameters related to these processes in AS and WT mice. We first observed an increase in 5-HT7R levels in AS homogenates as compared to WT, while 5-HT7R expression levels were significantly decreased in AS synaptosomes. These changes could reflect alteration in intracellular transport of 5-HT7Rs in AS neurons. In light of the well-known role of 5-HT7R in modulating structural and synaptic plasticity of mammalian brain circuits (Crispino et al., 2020; Ogelman et al., 2024; Olusakin et al., 2020; Volpicelli et al., 2014; Zareifopoulos and Papatheodoropoulos, 2016), these results suggest a selective involvement of this receptor in the molecular alterations at AS synapses. One of the mechanisms modulating synaptic plasticity consists in synaptic protein synthesis (Castillo et al., 2023; Di Giaimo et al., 2020; Perrone-Capano et al., 2021; Wong et al., 2024). Different classes of proteins have been demonstrated to be locally synthesized in axonal territory, including cytoskeletal proteins, such as neurofilament subunits and β-actin (Crispino et al., 2018, 1993; Leung et al., 2006; Ströhl et al., 2017) and proteins linked to synaptic vesicles such as Munc18–1 (Parvin et al., 2019) and SNAP25 (Batista et al., 2017). In addition, proteins of multiple functions such as the protease inhibitor cystatin B (Di Matteo et al., 2020; Penna et al., 2019; Rispoli et al., 2013), huntingtin (Shigeoka et al., 2016), and β-catenin (Taylor et al., 2013) are also locally synthesized. This variety of proteins being locally synthesized in nerve terminals emphasized the crucial role of synaptic protein synthesis in physiological events of the CNS. Interestingly, a growing number of data indicates that deregulation of this system is implicated in various CNS pathologies (Castillo et al., 2023; Cefaliello et al., 2020; Joo and Benavides, 2021; Perrone-Capano et al., 2021). Thus, we first focused on the synaptic translation system in AS mice cortex. Synaptic protein synthesis was significantly decreased in AS synaptosomes as compared to WT. Recent data indicated increased protein synthesis in the dorsal hippocampus of AS adult mice as compared to WT (Aria et al., 2023). The apparent discrepancy of these data with our results might be attributed to differential modulation of protein synthesis in different subcellular compartments in AS and WT mice. More importantly, incubation of AS synaptosomes with LP-211 led to a significant increase of local protein synthesis as compared to control, indicating that 5-HT7R stimulation can reverse, at least in part, the diminished synaptic protein synthesis of AS. The effect of LP-211 was different in WT synaptosomes, where a reduction in local protein synthesis was observed. This LP-211-dependent reduction of protein synthesis in synaptosomes from WT mice is quite intriguing when compared with the positive effect of 5-HT7R stimulation on protein synthesis in hippocampal neurons (Speranza et al., 2013). This different response, with a positive effect on the overall neuronal system of protein synthesis and an inhibitory effect at the synaptic level, emphasizes the selective action of LP-211 at synapses. It is important to note that the LP-211 effect in WT synaptosomes was not reversed by co-incubation with 5-HT7R antagonist, suggesting that additional molecular mechanisms may be involved, which are yet to be unveiled.

Synaptic plasticity mechanisms and their alterations in neuropathologies are tightly related to morphological and functional features of dendritic spines (Ebert and Greenberg, 2013; Heck and Santos, 2023; Khanal and Hotulainen, 2021; Lo and Lai, 2020). Several studies have found that AS neurons display a decrease in dendritic spine density in various brain areas (Dindot et al., 2007; Sun et al., 2015a; Yashiro et al., 2009). It is also well-known that 5-HT7R activation leads to an increase in the number of dendritic spines in hippocampal neuronal cultures and in the developing prefrontal cortex (Marin and Dityatev, 2017; Ogelman et al., 2024; Speranza et al., 2017). Therefore, we evaluated whether 5-HT7R activation of AS hippocampal neurons could reverse their dendritic spine abnormality. Prolonged stimulation of 5-HT7R with LP-211 resulted in an increase in dendritic spine density in AS neurons, which was partially reversed by the specific antagonist of 5-HT7R, SB-269970. LP-211 also induced an increase in dendritic spine density in WT hippocampal neurons, in line with previous reports on striatum and cortical neurons (Speranza et al., 2017). Interestingly, also in another neurodevelopmental disorder, Fragile X syndrome (FXS), an increase in dendritic spine density was observed, together with an elongated immature spines morphology (Irwin et al., 2001; Jawaid et al., 2018; Lee et al., 2019; Speranza et al., 2022). In general, atypical dendritic spine density and morphology characterize numerous neuropathologies, reflecting the underlying synaptic deficits (Penzes et al., 2013, 2011; Phillips and Pozzo-Miller, 2015). Thus, it is intriguing to hypothesize that stimulation of 5-HT7R, which restores dendritic density and morphology, could be an appropriate approach for different synaptopathies.

Recent findings revealed that the absence of UBE3A in AS hippocampal neurons, is associated with axonal guidance deficit and increased axonal branching, due to altered cytoskeleton dynamics (Tonazzini et al., 2019). It has been established that the axonal outgrowth induced by LP-211 treatment, depends on actin dynamics through mTOR pathway activation (Speranza et al., 2015). Interestingly, the mTOR pathway is also implicated in AS pathology (Sun et al., 2016, 2015b). Therefore, it is possible to hypothesize that mTOR is one of the pathways involved in the spine density rescuing effect of LP-211 in AS neurons.

To extend our analysis to the electrophysiological level, we investigated the possible rescuing effect of 5-HT7R stimulation on LTP, a plasticity mechanism impaired in AS mice (Baudry et al., 2012) and previously found to be modulated by 5-HT7Rs (Khodaverdi et al., 2021; Lippiello et al., 2016; Roberts et al., 2004). Our results clearly showed that 5-HT7R stimulation with LP-211 could reverse the LTP impairment in AS mice, strongly suggesting a crucial role of 5-HT7R also in synaptic function and modulation.

Since LTP is a crucial mechanism underlying learning and memory, we investigated the effects of 5-HT7R stimulation on learning and memory in AS mice. As previously reported (Baudry et al., 2012; Sun et al., 2016, 2015a), AS mice exhibited a deficit of long-term memory when compared to WT in the fear conditioning paradigm, as indicated by the reduction in freezing scores to the context and to the tone. Treatment with LP-211 selectively rescued the behavioural impairment in contextual fear conditioning but did not have a significant effect on tone fear conditioning. The contextual fear conditioning depends on a neural circuit including the hippocampus, basolateral amygdala and medial prefrontal cortex (Maren et al., 1998), while tone fear conditioning appears to be hippocampus-independent (Phillips and LeDoux, 1994). Although 5-HT7Rs are similarly distributed in different brain regions, including the amygdala and the hippocampus (Gustafson et al., 1996; Neumaier et al., 2001; Varnäs et al., 2004), data on the effect of its stimulation on fear conditioning responses are somewhat controversial. In particular, 5-HT7R knock-out mice exhibit deficits in contextual but not in cued fear conditioning (Roberts et al., 2004), a result in agreement with our data. On the other hand, administration of a 5-HT7R selective antagonist was shown to cause a significant decrease in both context- and tone-dependent fear conditioning (Matsushita et al., 2009). While these results confirm the role played by 5-HT7R signalling in modulating behavioural responses, further investigations are necessary to unveil the underlying molecular mechanisms in different brain regions.

## 5. Conclusions

Our data, showing impairment of local protein synthesis in synapses in AS mice, extend the knowledge on the molecular mechanisms by which synaptic plasticity is altered in this genetic disorder. Furthermore, we showed that activation of serotonergic signalling pathways could ameliorate the molecular, morphological and cognitive deficits displayed by AS mice. Altogether, these data suggest that 5-HT7R could represent a promising target for pharmacological treatment of the disease.

## Data Availability

Data will be made available on request.
